# Overexpression of cotton *GhNAC072* gene enhances drought and salt stress tolerance in transgenic Arabidopsis

**DOI:** 10.1186/s12864-022-08876-z

**Published:** 2022-09-12

**Authors:** Teame Gereziher Mehari, Yuqing Hou, Yanchao Xu, Muhammad Jawad Umer, Margaret Linyerera Shiraku, Yuhong Wang, Heng Wang, Renhai Peng, Yangyang Wei, Xiaoyan Cai, Zhongli Zhou, Fang Liu

**Affiliations:** 1 State Key Laboratory of Cotton Biology, Cotton Institute of the Chinese Academy of Agricultural Sciences, Anyang, China; 2 School of Life Sciences, Nantong University, Nantong, 226019 Jiangsu China; 3 Anyang Institute of Technology, Anyang, Henan China; 4 School of Agricultural Sciences, Zhengzhou University, Zhengzhou, China

**Keywords:** Cotton, Drought, Salt, *GhNAC072*, Transcription factor, Overexpression

## Abstract

**Background:**

Crops face several environmental stresses (biotic and abiotic), thus resulting in severe yield losses. Around the globe abiotic stresses are the main contributors of plant damages, primarily drought and salinity. Many genes and transcription factors are involved in abiotic and biotic stress responses. *NAC* TF (Transcription Factors) improves tolerance to stresses by controlling the physiological and enzyme activities of crops.

**Results:**

In current research, *GhNAC072* a highly upregulated TF in RNA-Seq was identified as a hub gene in the co-expression network analysis (WGCNA). This gene was transformed to *Arabidopsis thaliana* to confirm its potential role in drought and salt stress tolerance. Significant variations were observed in the morpho-physiological traits with high relative leaf water contents, chlorophyll contents, higher germination and longer root lengths of the overexpressed lines and low excised leaf loss and ion leakage as compared to the wildtype plants. Besides, overexpressed lines have higher amounts of antioxidants and low oxidant enzyme activities than the wildtype during the period of stress exposure.

**Conclusions:**

In summary, the above analysis showed that *GhNAC072* might be the true candidate involved in boosting tolerance mechanisms under drought and salinity stress.

**Supplementary Information:**

The online version contains supplementary material available at 10.1186/s12864-022-08876-z.

## Background

Crops play a vital part in our lives but, face several environmental stresses regularly. The plant perceives and transduces the stress signals, which leads to the expression of functional proteins that safeguard the plant. Genes that respond to stresses are primarily controlled by transcription factors, which are vital in plant resistance to the changing environmental conditions [1]. A transcription factor (TF) is a type of important regulatory protein that regulates the expression level of a gene [2]. It governs chromatin and transcription by recognizing certain DNA sequences, forming a complex mechanism that directs genome expression [3].

Abiotic stress is the harmful influence of abiotic factors on living things in a definite location. Drought, high temperature, cold, salt stress and other ecological extremes are among the stresses. Crop losses are primarily caused by salinity and drought around the globe [4]. Plant cells produce a huge quantity of reactive oxygen species (ROS) when they are under stress. Under these circumstances, the activity of superoxide dismutase (SOD), catalase (CAT), and peroxidase (POD) enzymes will rise to some extent to eliminate toxic compounds from the plant and preserve normal development [5].

Plant transcription factors include more than 80 different TF families. *NAC*, *MYB*, *WRKY*, *bZIP*, and *ERF*/*DREB* are only a few TFs that are involved in abiotic and biotic stress responses [6]. The NAC TF is part of one of the biggest families of TFs involved in crops response to abiotic stresses [7]. A *NAC* TF has a two way regulatory approach: The first is transcriptional regulation, which includes phosphorylation and ubiquitination miRNAs, which can control TF expression at the protein level. The alternative method is to attach to the target mRNA and perform posttranscriptional regulation. *NAC* TF can impact the final output by binding to sequences and regulating target genes [1]. *NAC* TF improved tolerance to stress by controlling the physiological and enzyme activities of crops. So far, this family has been studied in a wide range of crops. In *Malus baccata*, the transformation of the *MbNAC25* gene in Arabidopsis improves cold and salinity tolerance [1]. Drought tolerance was dramatically increased and lowered in transgenic rice plants via overexpressing and RNA interfering in the *OsDRAP1* transcription factor [8].

Transformation of *ZmNAC33* transcription factor from maize was done in *A. thaliana*. The germination percentage under ABA and osmotic stress during the germination stage are higher than that of wildtype seedlings. Compared with the low wild-type drought stress, the overexpression lines show a higher survival rate and higher antioxidant enzyme activity [9]. *JUNGBRUNNEN1NAC* TF improved drought tolerance in *Solanum lycopersicum* [10]. The *mOsNAC2* overexpressing lines were much more drought and salinity tolerant than the wildtype, with a higher survival rate thanks to higher ABA levels [11]. Similar research finding in rice indicated that the *ONAC022oe* plants had lower rates of water loss and transpiration, a lower proportion of opening stomata collected fewer Na^+^ in roots, and had higher levels of proline and soluble carbohydrates than wildtype seedlings during drought and salinity stress [12]. Under drought and salt stress, overexpressed lines had higher expressions of *NtARF1*, *NtARF2*, and *NtARF8*, implying that overexpression of *SlNAC35* aided root growing by engaging auxin signaling and modulating *NtARF* expression in tomato [13]. The ABA-dependent signaling system improves drought and cold stress tolerance in overexpressed lines by transforming the *MLNAC5* gene from *Miscanthus lutarioriparius* as a stress responsive *NAC* TF [14].

Several experiments was done specifically on *NAC072* TF in different crop species. A NAC TF from *Oxytropis ochrocephala*, *OoNAC72* enhanced drought, salt stress tolerance, and ABA-dependent process regulation in Arabidopsis. *OoNAC72* overexpression also increased the expression of stress-responsive genes like *RD29A*, *RD29B*, *RD26*, *LEA14*, *ANACOR19*, *ZAT10*, *PP2CA*, and *NCED3* [15]. In line with the above finding, overexpression of *RcNAC72*, a NAC TF from *Rosa chinensis*, improved drought tolerance and ABA sensitivity in Arabidopsis. Furthermore, in rose leaves, silencing *RcNAC72* decreases tolerance to water loss stress and rehydration. The regulation mechanism of *RcNAC72* via the ABA-dependent signaling pathway and the *DRE*/*CBF-COR* pathway in response to drought stress identified [16]. There is an interaction between *NAC072* and ABA-responsive element binding factor 3 (*ABF3*) in Arabidopsis, which acts as a positive regulator of ABA-responsive gene expression. *ABF3* upregulates *NAC072* expression in the ABA response, and *NAC072* protein also interacts with *ABF3* [17].

Transcriptome analysis in cotton species was very crucial for mining of genes involved in abiotic and biotic stresses. RNA-Seq analysis was done to identify differentially expressed genes in cadmium stress tolerance [18], under NaHCO_3_ alkaline stress [19], in cotton boll weevil pests [20], in *Aspergillus flavus* fungi responsible for Aflatoxin production [21], in fiber growth and development [22], in anther development and male sterility traits [23], and DEGs linked to Na_2_SO_4_ tolerance [24]. To better understand the role of the *NAC* genes in drought and salinity stress, we cloned the *NAC* transcription factor gene *GhNAC072* from *G. hirsutum* L. The function of this TF was investigated and recognized as a potential candidate for improved cotton cultivations under drought and salt stress.

## Results

### Identification of *NAC *genes in *G. hirsutum, G. arboreum and G. raimondii*

**Fig. 1:**
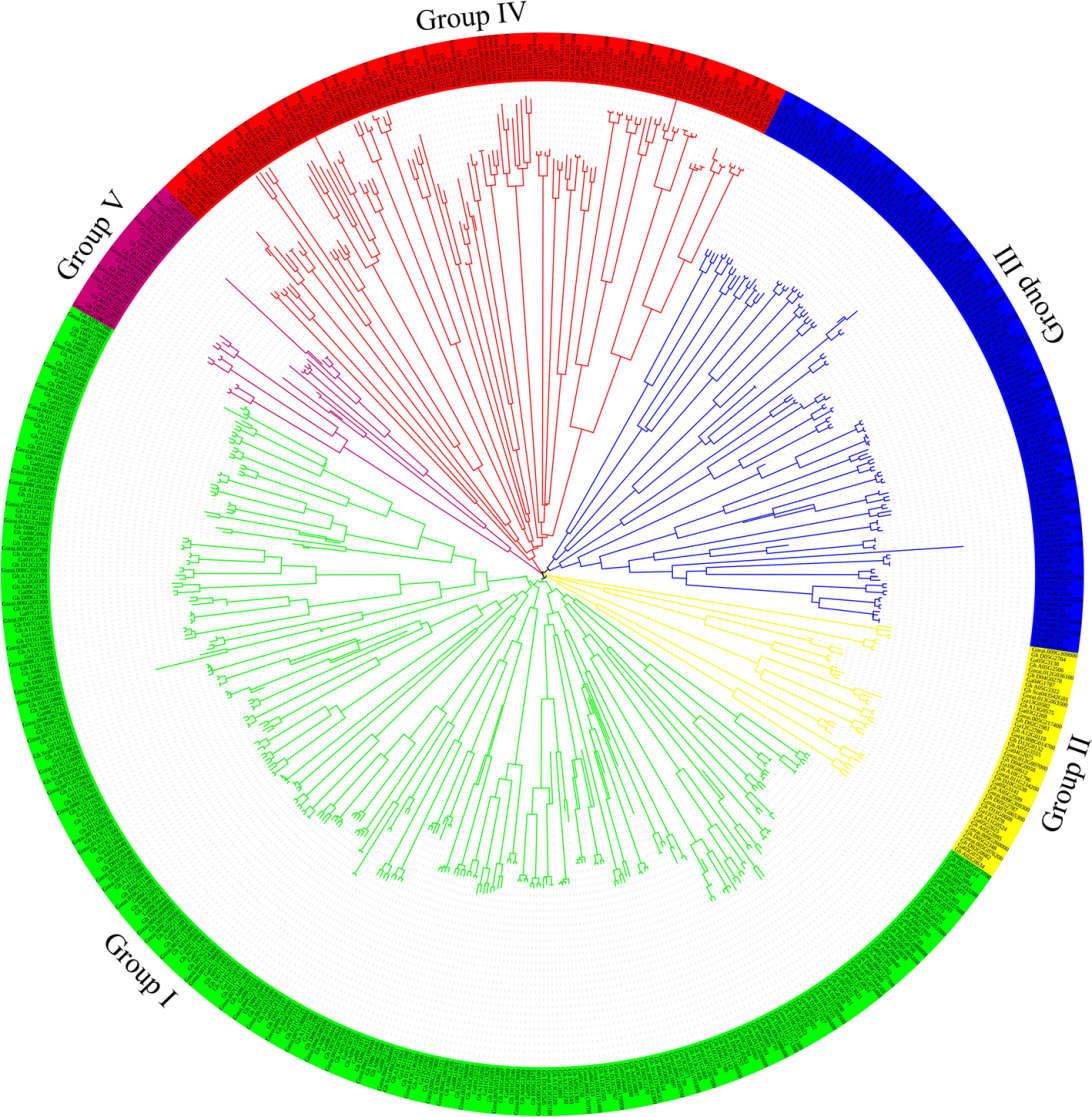
Phylogenetic analysis of the NAC proteins from *G. hirsutum, G. arboreum,* and *G. raimondii*. The phylogenetic tree was constructed by iTOL online tool, with ClustalX alignment. The evolutionary tree was divided the species in to five groups, each color represents one group

We identified a total of 608 *NAC* TF in the three *Gossypium* species, 305 in *G. hirsutum* with 152 genes in GhAt subgenome, 144 genes in GhDt subgenome and 9 genes in scaffold region, 150 in *G. arboreum* and 153 in *G. raimondii* according to CottonFGD (www.cottonfgd.org) database. 295, 44, 123, 119 and 27 NAC genes were found in group I, Group II, Group III, Group IV and Group V respectively. The highest number of genes were found in group I with 153 NAC genes in *G. hirsutum*, 72 in *G. arboreum* and 70 in *G. raimondii* while the lowest scored in group V with 13, 7 and 7 NAC genes in *G hirsutum*, *G. arboreum* and *G. raimondii* respectively (Table S1), (Fig. 1).

### Chromosomal mapping and gene ontology analysis

Genome wide analysis of the three *Gossypium* species revealed that, the *NAC* TF were distributed among all thirteen chromosomes. Both At05 and At11 chromosomes has the maximum number of genes (15 genes) from the At subgenome individually, whereas the lowest number of genes (6 genes) were located on chromosomes At04 and At06. Similarly, Dt11 and Dt05 had the most gene loci with 15 and 12 genes in the Dt Subgenome, whereas Dt03, Dt08, and Dt10 had 7, 7, and 5 genes, respectively. The remaining chromosomes had 6 and 15 genes on them. The gene distribution arrangement was similar among two diploid *Gossypium* species, *G. arboreum* and *G. raimondii*. The highest gene loci in *G. arboreum* were found on chromosomes A11 and A05, with 17 and 15 genes, respectively, whereas in *G. raimondii*, the highest gene loci were recorded on chromosomes D07 and D09, with 17 and 16 genes, respectively, while chromosomes D02, D03, and D13 had 3, 7 and 9 genes, respectively. The scaffold category harbored 33 *NAC* genes together (Fig S1).

There was an odd distribution of genes between the chromosomes of *G. hirsutum* and both diploid species, *G. arboreum* and *G. raimondii*. In *G. hirsutum* At subgenome and *G. arboreum* 5^th^ and 9^th^chromosomes showed the same number of genes with 15 and 9 genes respectively. Similarly, chromosomes 2, 3, and 5 harbor the same number of genes in the GhDt subgenome and *G. raimondii* with 11, 7, and 14 genes respectively. Only six chromosomal pairs from the GhAt/A and GhDt/D contrasts were discovered to have a similar number of genes (Table 1). This depicts the rate of gene loss during evolution, as well as the role of progenitors in the development of tetraploid cotton species [25]. The unequal distribution of genes on the cotton genome's chromosome could be due to gene replication or a partial segment of gene duplication that happened during the plant's long evolutionary history. With each duplication, cotton's whole gene sequence was doubled, and the extra genes were recombined or eliminated with time [26].

**Table 1:** Distribution of genes on the chromosomes of three *Gossypium* species

*G. hirsutum* (GhAt) Vs *G. arboreum* (Ga)				*G. hirsutum* (GhDt) Vs *G. raimondii* (Gr)			
**Chr. No**	**Genes**	**Chr. No**	**Genes**	**Chr. No**	**Genes**	**Chr. No**	**Genes**
GhAt-01	10	GaA-01	13	GhDt-01	8	GrD-01	10
GhAt-02	8	GaA-02	3	GhDt-02	11	GrD-02	11
GhAt-03	8	GaA-03	10	GhDt-03	7	GrD-03	7
GhAt-04	6	GaA-04	9	GhDt-04	10	GrD-04	15
GhAt-05	15	GaA-05	15	GhDt-05	14	GrD-05	14
GhAt-06	6	GaA-06	9	GhDt-06	9	GrD-06	13
GhAt-07	10	GaA-07	9	GhDt-07	10	GrD-07	17
GhAt-08	13	GaA-08	15	GhDt-08	11	GrD-08	13
GhAt-09	11	GaA-09	13	GhDt-09	12	GrD-09	16
GhAt-10	9	GaA-10	9	GhDt-10	5	GrD-10	10
GhAt-11	15	GaA-11	17	GhDt-11	15	GrD-11	10
GhAt-12	12	GaA-12	13	GhDt-12	11	GrD-12	8
GhAt-13	10	GaA-13	8	GhDt-13	7	GrD-13	9
Scaffold	13	Scaffold	5	Scaffold	11	Scaffold	0

The Gene Ontology (GO) project was a large bioinformatics endeavor that aimed to create a computer description of how genes encode activities at the biological, molecular, and cellular system levels [27]. In *G. hirsutum*, the biological function (GO:0,008,150) includes biological regulation, regulation of biological process, cellular process, and metabolic process that regulates transcription and DNA-dependent functions. Similarly, in molecular function—GO:0,003,674, the significant role was binding of nucleic acid and DNA functions (Fig S2).

In both diploid species the significant functions were similar to *G. hirsutum*, biological and molecular functions. In *G. arboreum* the biological process function—GO:0,008,150 deals with metabolic process, biological regulation, regulation of biological process, cellular process, developmental process, single-organism process, multicellular organismal process, reproduction, cellular component organization or biogenesis and response to stimulus functions. In molecular activity—GO:0,003,674, binding and nucleic acid binding transcription factor activity functions. For *G. raimondii*, the biological process—GO:0,008,150 mainly on metabolic process, cellular process, biological regulation, and regulation of biological process functions. In molecular function—GO:0,003,674, Binding which regulates organic cyclic compound binding and heterocyclic compound binding that leads to nucleic acid binding for final DNA binding processes. In all the three cotton species, the molecular function was similarly related to the binding of nucleic acids and there is no significant cellular function in the GO category.

### Gene structure and motif identification analysis

In gene structure analysis of NAC TFs, high variability was found between all genes. In *G. hirsutum*, the highest exon–intron ratios were scored for *Gh_D02G0122* with 9 exons and 8 introns, whereas just one exon was present in, *Gh_A01G0806, Gh_A01G0131,Gh_D11G0608, Gh_A11G0524, Gh_Sca077544G01, Gh_Sca121194G01, Gh_Sca111532G01, Gh_Sca140422G01, Gh_Sca043542G01* and *Gh_Sca083050G01* genes (Fig S1 A).

In *G. arboreum*, *Ga03G0113* has the highest numbers of exons and introns (14 exons, 13 introns) followed by *Ga12G0369* (9 exons, 8 introns) whereas, the lowest exon–intron ratio of 2:1 was found in *Ga09G2767, Ga01G0440, Ga14G2861, Ga06G2355,* and *Ga13G0670* genes. Similarly, In *G. raimondii Gorai.008G261400* (9 exons, 8 introns), possesses the highest number of exons and introns, followed by *Gorai.005G013300, Gorai.007G114500*, and *Gorai.006G034600* with (7 exons, 8 introns), while a single exon was found in *Gorai.002G019700* (Fig S3 C).

To better understand the structural evolution of *NAC* proteins, the patterns of motifs were investigated. In the three *Gossypium* species, MEME analysis (http://meme-suite.org/) showed a total of 20 unique motifs. Based on the known motifs, In *G. hirsutum* motifs 3, 4, 1, 7, 2, and 5 were conserved while motifs 6, 14, 15, and 17 were diversified once. In *G. arboreum,* motifs 2, 5, 1, 4, 3, 6, and 8 were conserved with 16, 18, 19, and 20 diversified. Similarly, in *G. raimondii*, motifs 3, 4, 1, 12, 2, 6, and 5 were conserved, however, 17, 18, 19, and 20 were found as diversified motifs. All three cotton species shared almost the same conserved and diversified motifs (Fig S3).

### Co-expression network analysis of *NAC* TFs for hub gene identification

**Fig. 2:**
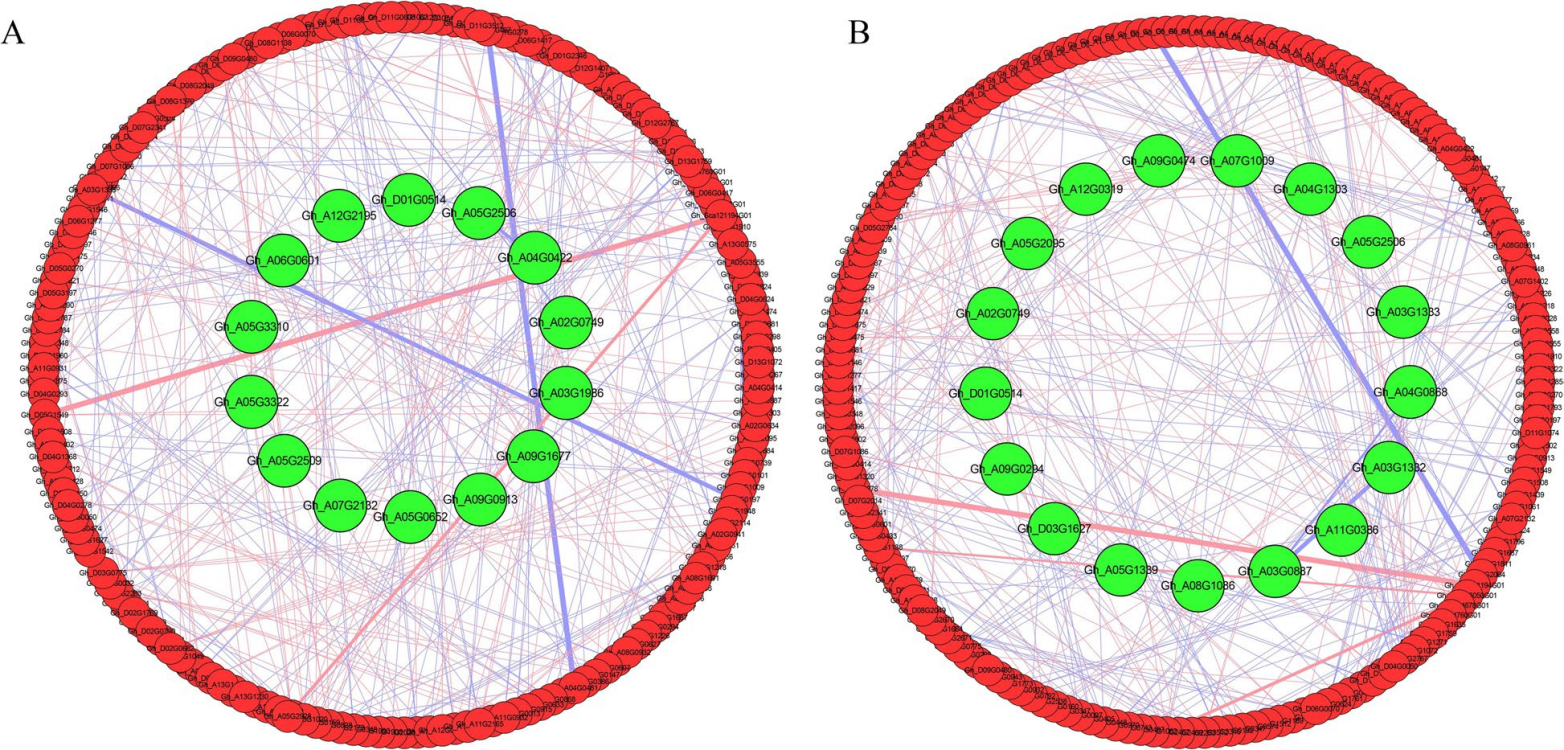
The Pearson correlation network analysis reveals drought tolerance of NAC genes **A** Co-expression network of genes in Leaves, **B** Co-expression network of genes in Roots. Genes in the middle of the network with green light represent hub genes. Red line represents positive correlation and blue line represents negative correlation. The thickness of individual line denotes the value of the correlation coefficient for individual connected pairs

Co-expression network analysis was performed using 193 DEGs selected from the RNA-Seq of *Gossypium hirsutum* races related to drought stress tolerance. Correlation based relationships were plotted using a Pearson correlation coefficient greater than 0.99 (Fig. 2). The network has a total of 193 nodes connected in it, with 1768 edges in the leaves and 1801 in the roots. In case of leaves we found, 973 positive and 795 negative correlations, whereas in roots 993 were positive and 808 were negatively correlated. A threshold level of > 20 edges was considered as hub genes from the analysis. Owing to this, 14 genes in leaf tissues and 17 in the roots were considered as hub genes. Among the hub genes, highest correlation was recorded in *Gh_A05G3322* with 27 and *Gh_A03G0887* scored 29 correlations in leaf and root tissues respectively. The candidate gene *Gh_D01G0514* (*GhNAC072*) was included in both correlations as a hub gene.

The *GhNAC072* gene has a protein length of 346 aa and a CDS length of 1041 bp. There are 3 exons, 2 introns in *GhNAC072* gene with a molecular weight of 38.4 KDa, and an isoelectric point of 9, with -0.573 grand average of hydropathy values (Table 2).

**Table 2:** Characterization of *Gh_D01G0514* (*GhNAC072*)

Gene features	Gene Name	Chromosome	Start	End	Length (bp)
	*GhNAC072*	D01	6,360,473	6,361,691	1,219
Transcript features	CDS Length (bp)	CDS GC Content (%)	Exon Number	Mean Exon Length (bp)	Mean Intron Length (bp)
	1041	43.3	3	347	89
Protein statistics	Protein Length (aa)	Molecular Weight (kDa)	Charge	Isoelectric Point	Grand Average of Hydropathy
	346	38.436	7	9.009	-0.573

### Expression of NAC genes in leaves and roots under drought and salinity stress

**Fig. 3:**
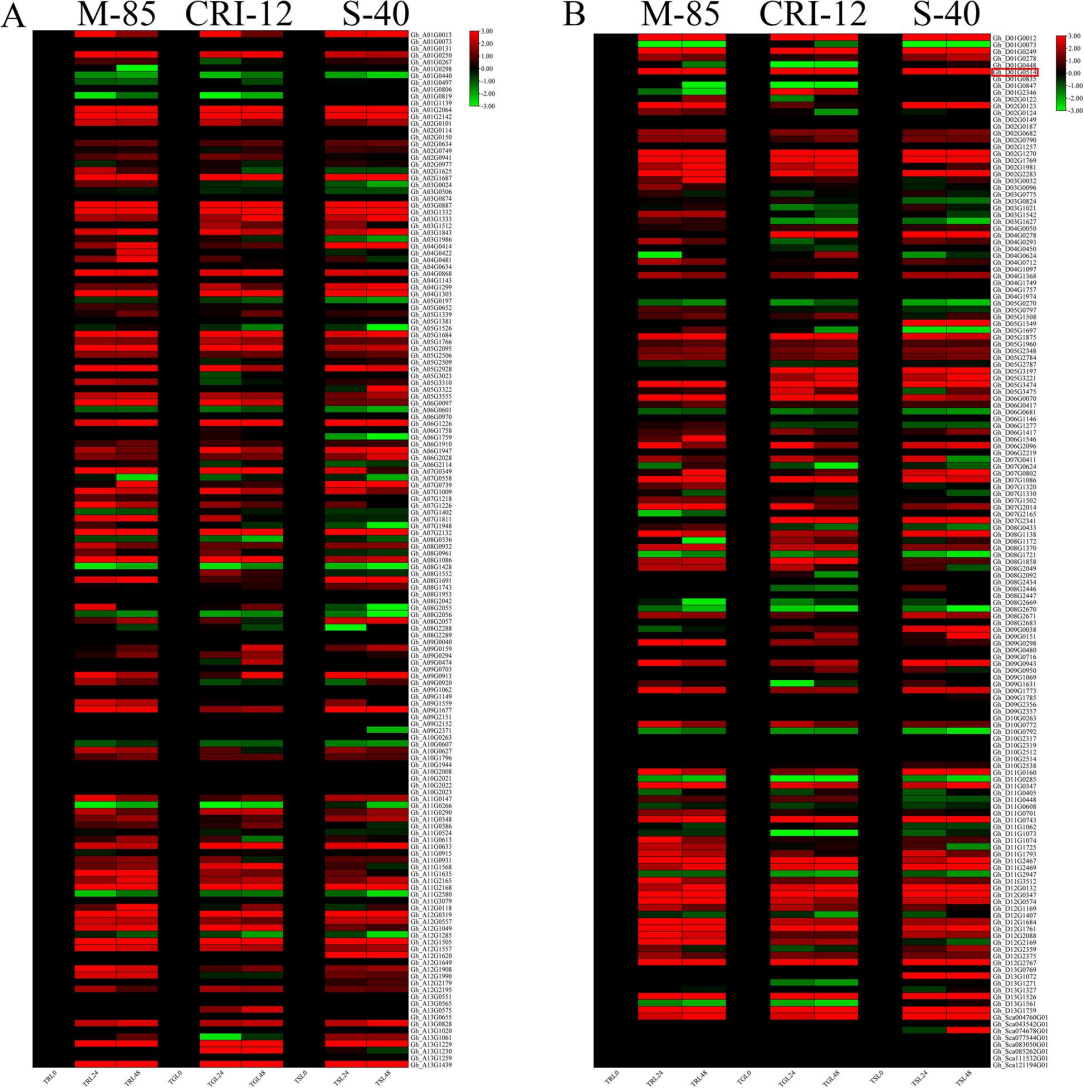
**A** Expression analysis of NAC genes in *G. hirsutum* At subgenome in leaf tissues of three *G. hirsutum* lines, Mariegalante-85, Upland cotton and Latifolium-40 under drought stress **B** Expression analysis of NAC genes in *G. hirsutum* Dt subgenome in leaf tissues of three *G. hirsutum* lines, Mariegalante-85, Upland cotton and Latifolium-40 under drought stress. Three biological replication was kept and Log-twofold change was applied for data normalization

**Fig. 4:**
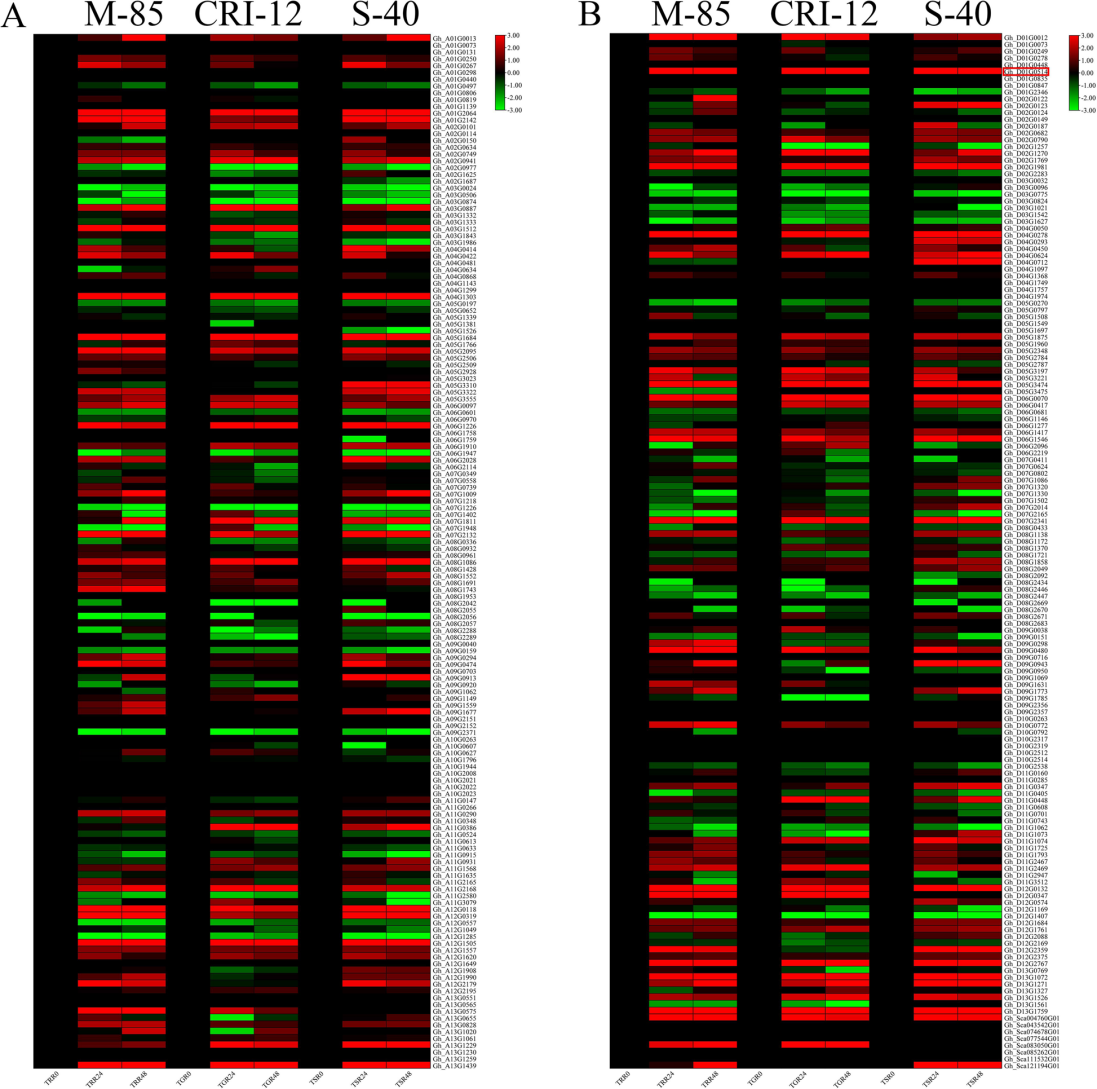
**A** Expression analysis of NAC genes in *G. hirsutum* At subgenome in root tissues of three *G. hirsutum* lines, Mariegalante-85, Upland cotton and Latifolium-40 under drought stress **B** Expression analysis of NAC genes in *G. hirsutum* Dt subgenome in root tissues of three *G. hirsutum* lines, Mariegalante-85, Upland cotton and Latifolium-40 under drought stress. Three biological replication was kept and Log-twofold change was applied for data normalization

Already available RNA-Seq data (PRJNA663204) of three cotton lines from *G. hirsutum* was used to detect the expression variations of NAC genes in leaf and root tissues at different time intervals. The *GhNAC* genes exhibited differential expression patterns in both tissues. According to the expression analysis most of *GhNAC* genes were upregulated in leaf tissues than roots tissues collected from the three cotton lines. There were no significant variations observed from 24 to 48 h. *GhNAC072*which was also selected as a hub gene via WGCNA analysis showed an upregulated expression in leaf tissues of all *Gossypium* species (Fig. 3, Fig. 4B).

Similarly, the RNA-Seq analysis of *NAC* genes also showed consistent expressions in Mariegalante-85, Upland cotton, and Latifolium-40 under drought stress in roots tissues. The gene expression profile in both tissues leads to selection of *Gh_D01G0514* as a candidate gene and increased our confidence (Fig. 4A).

### Selection of overexpressed lines from T2 generation

**Fig. 5:**
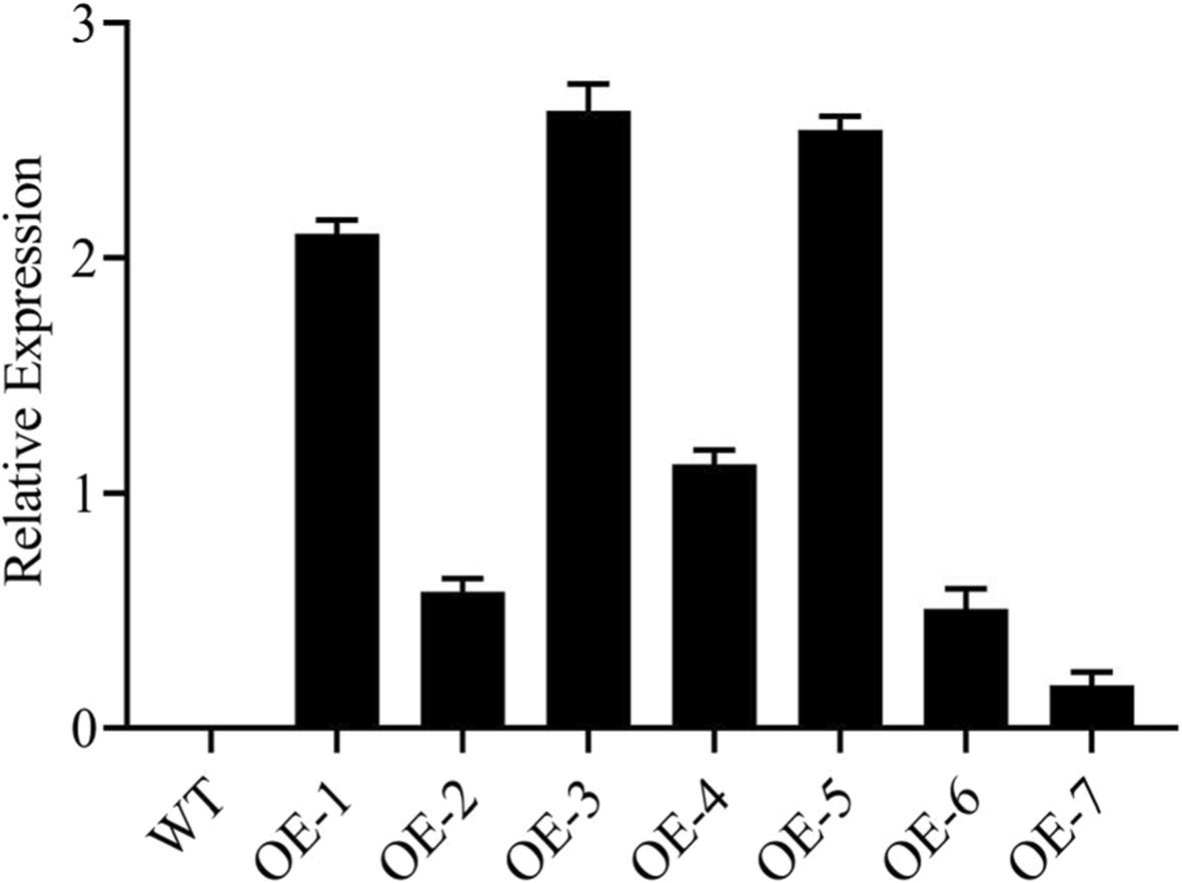
Relative expression of the overexpressed lines using RT-qPCR analysis in three technical and biological replications at T-2 generation

After transformation, we found seven overexpressed positive lines at the T2 generation stage. We performed PCR and RT-qPCR analysis to check the expression levels of all the lines. OE-1, OE-3, and OE-5 overexpressed lines were selected for further experiments due to their highest expression levels (Fig. 5).

### Evaluation of physiological parameters under drought and salt stress

**Fig. 6:**
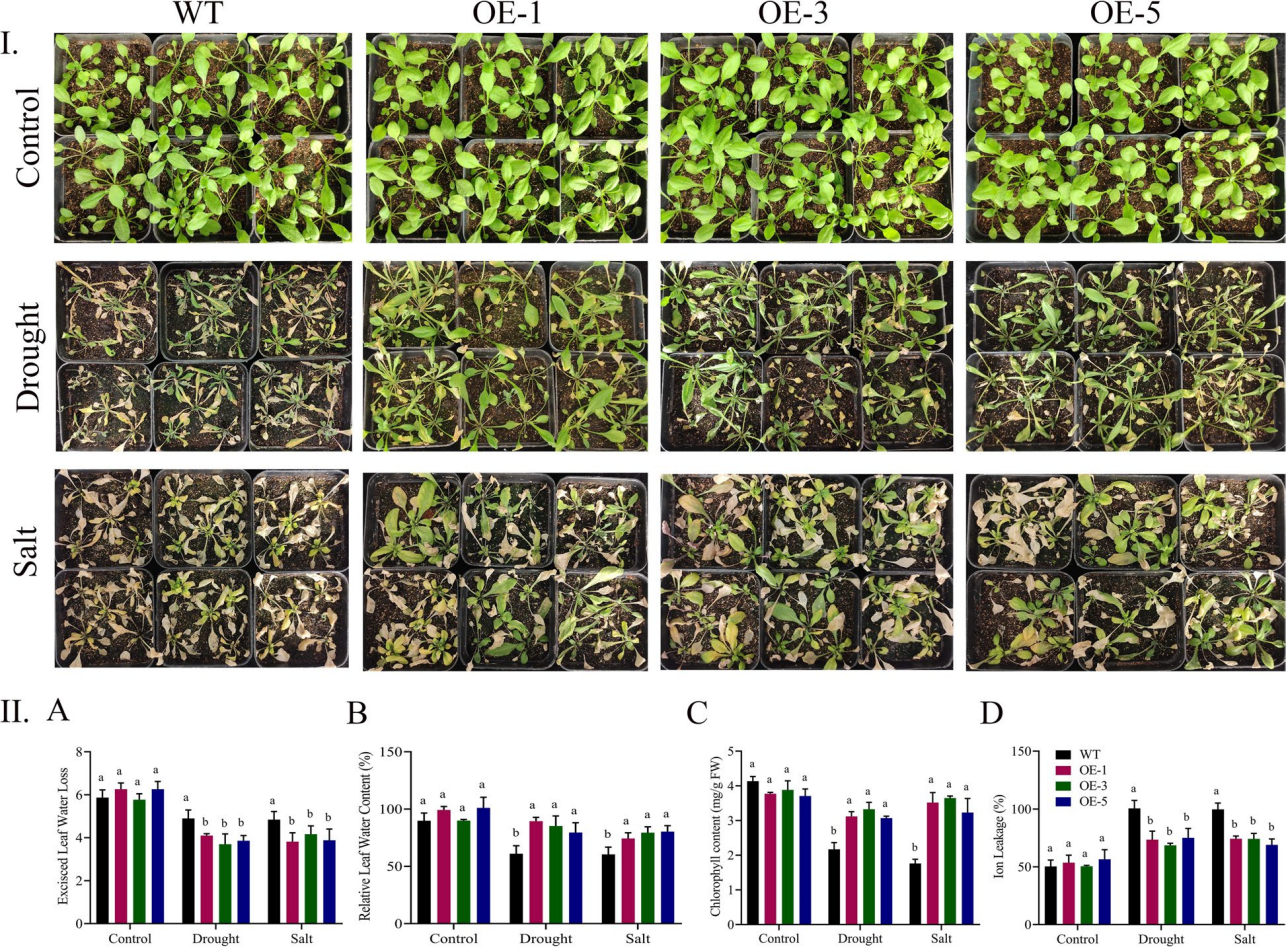
**I** Figurative illustration of the wildtype and overexpressed lines during normal, drought, and salinity conditions, **II** Physiological trait evaluation under drought and salinity settings **A** ELWL **B** RLWC **C** Chlorophyll content **D** Ion leakage, every experiment had three biological replications., means were calculated using LSD at the *P* < 0.05 and *P* < 0.01 confidence interval

A significant variation (*p* < 0.05) was observed among wildtype and overexpressed lines in excised leaf water loss. No significant variations were observed under control conditions, whereas, after drought and salt application, the wildtype seedlings had the highest leaf water loss and ion leakage as compared to the overexpressed lines. Significant differences (*P* < 0.05) were observed for relative leaf water contents and chlorophyll contents between the wildtype and overexpressed lines (Fig. 6).

### Determination of oxidant and antioxidant enzymes activities

**Fig. 7:**
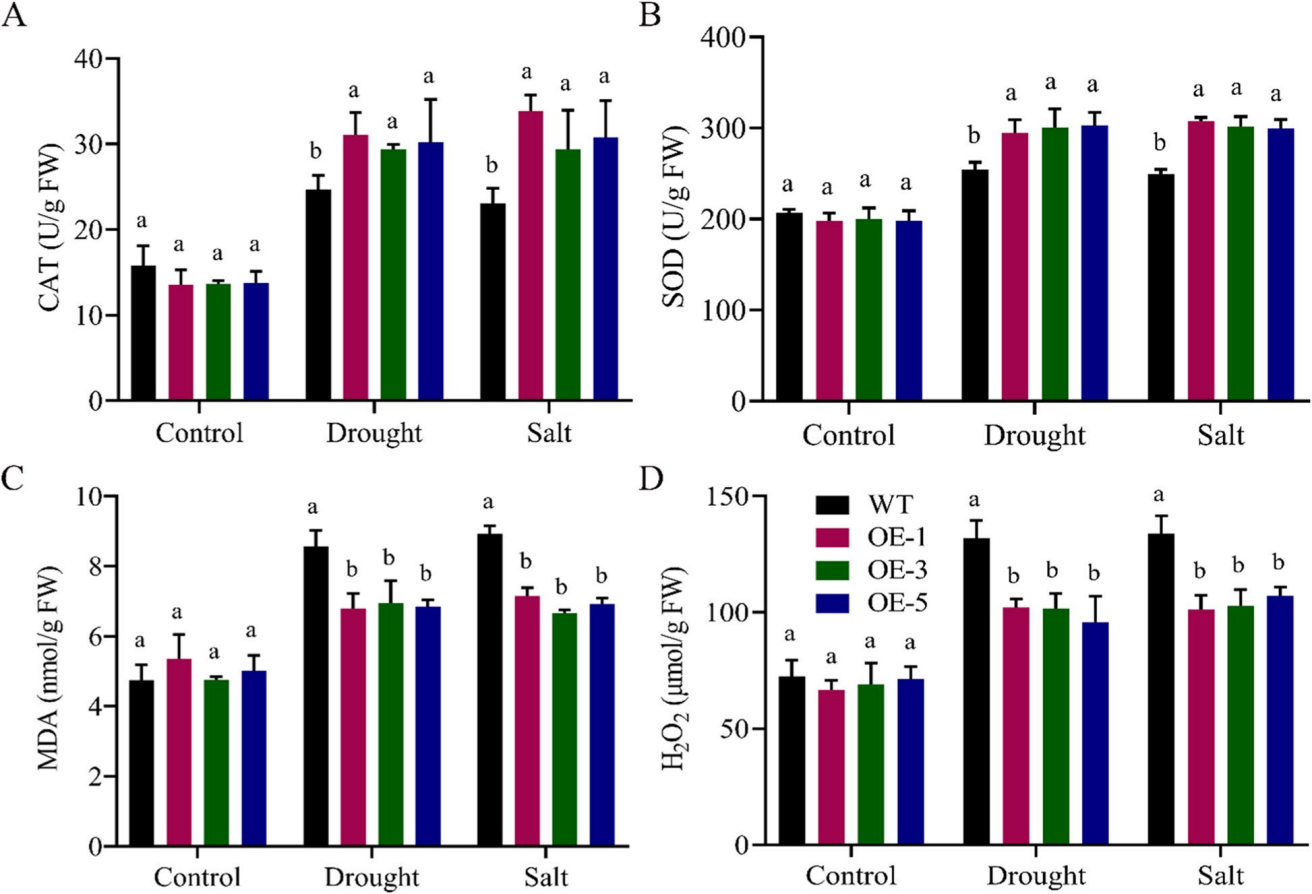
Antioxidants and oxidant enzymes determination under drought and salt stress conditions **A** Catalase enzyme **B** Super oxidase enzyme **C** Malondialdehyde enzyme **D** Hydrogen peroxide enzyme, every experiment had three biological replications., means were determined using LSD at the *P* < 0.05 and *P* < 0.01 confidence interval

Our results showed that the enzyme activities have been significantly influenced by drought and salt treatments. The activities of CAT and SOD were observed highest in the overexpressed lines than the wildtype. Whereas, the levels of oxidant enzymes, MDA and H_2_O_2_ were lower in the overexpressed lines than the wildtype under drought and salinity stress. Current results confirmed that overexpressed lines face a minimal damage as compared to wildtype under stress conditions (Fig. 7).

### *GhNAC072* enhances germination rate and root length in overexpressed lines

**Fig. 8:**
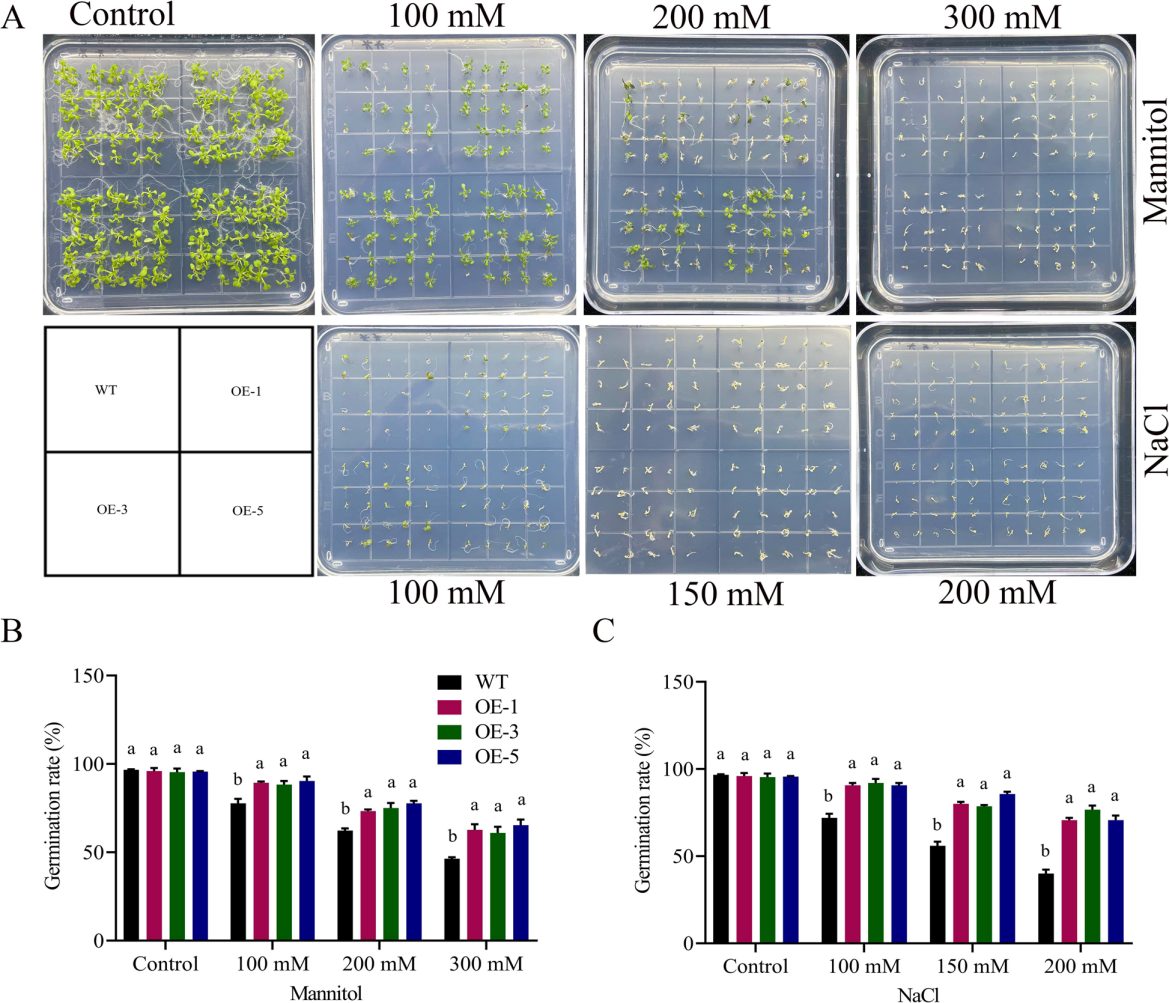
Germination percentage evaluation **A** Figurative illustration for germination percentage of wildtype and overexpressed lines in normal, drought, and salinity settings, **B** germination percentage determination in free MS media, MS media with 100 mM, 200 mM, and 300 mM mannitol, **C** Germination percentage determination in free MS media, MS media having 100 mM, 150 mM and 200 mM NaCl

Significant variations (*P* < 0.05) were observed for germination percentage in both, wildtype and overexpressed lines during drought and salinity stress conditions. The germination percentage of the overexpressed lines was higher than that of wildtype under 100 mM, 200 mM, and 300 mM mannitol concentrations. Under 300 mM mannitol concentration, the germination percentages of < 47% and > 67% were observed in case of wildtype and overexpressed lines respectively (Fig. 8). Similarly, under 200 mM NaCl concentration, the germination rate in wildtype was 40% but for overexpressed lines more than 72% germination rate was recorded in the current study.

**Fig. 9:**
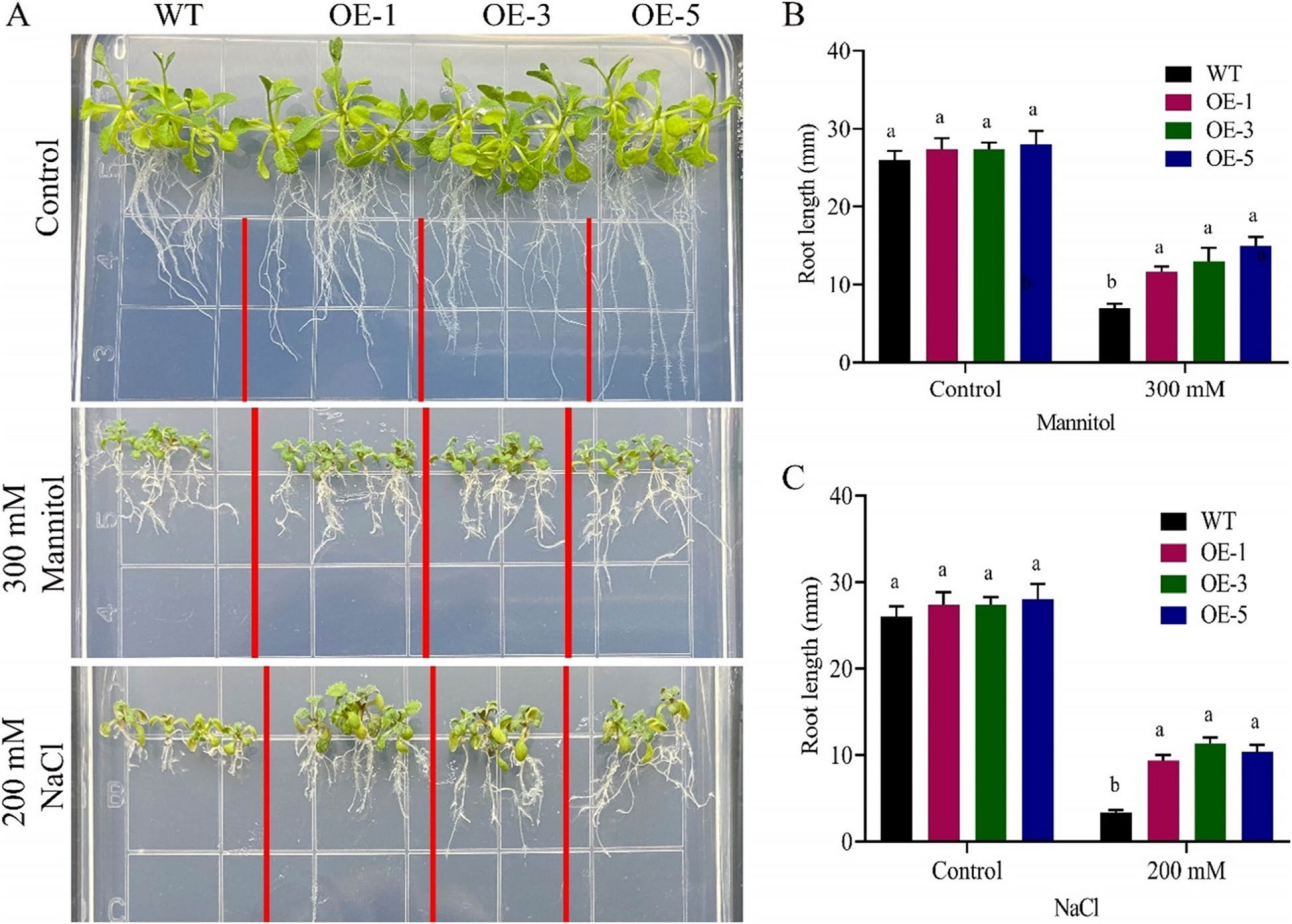
Root length determination of wildtype and overexpressed lines in drought and salinity treatment **A** Figurative illustration for root length of wildtype and overexpressed lines in normal, drought and salinity settings, **B** Root length determination in free MS media and MS media having 300 mM mannitol, **C** Root length determination in free MS media and MS media with 200 mM NaCl

Drought and salt stress treatments have a considerable impact on root length. There were no significant differences in root length between wildtype and overexpressed lines under normal circumstances. The overexpressed lines, on the other hand, had longer roots per line under both drought and salt treatments with 300 mM mannitol and 200 mM NaCl, respectively. This confirms that *GhNAC072*plays a major role in drought and salinity stress tolerance (Fig. 9).

### Evaluation of abiotic stress responsive genes under drought and salt stress

**Fig. 10:**
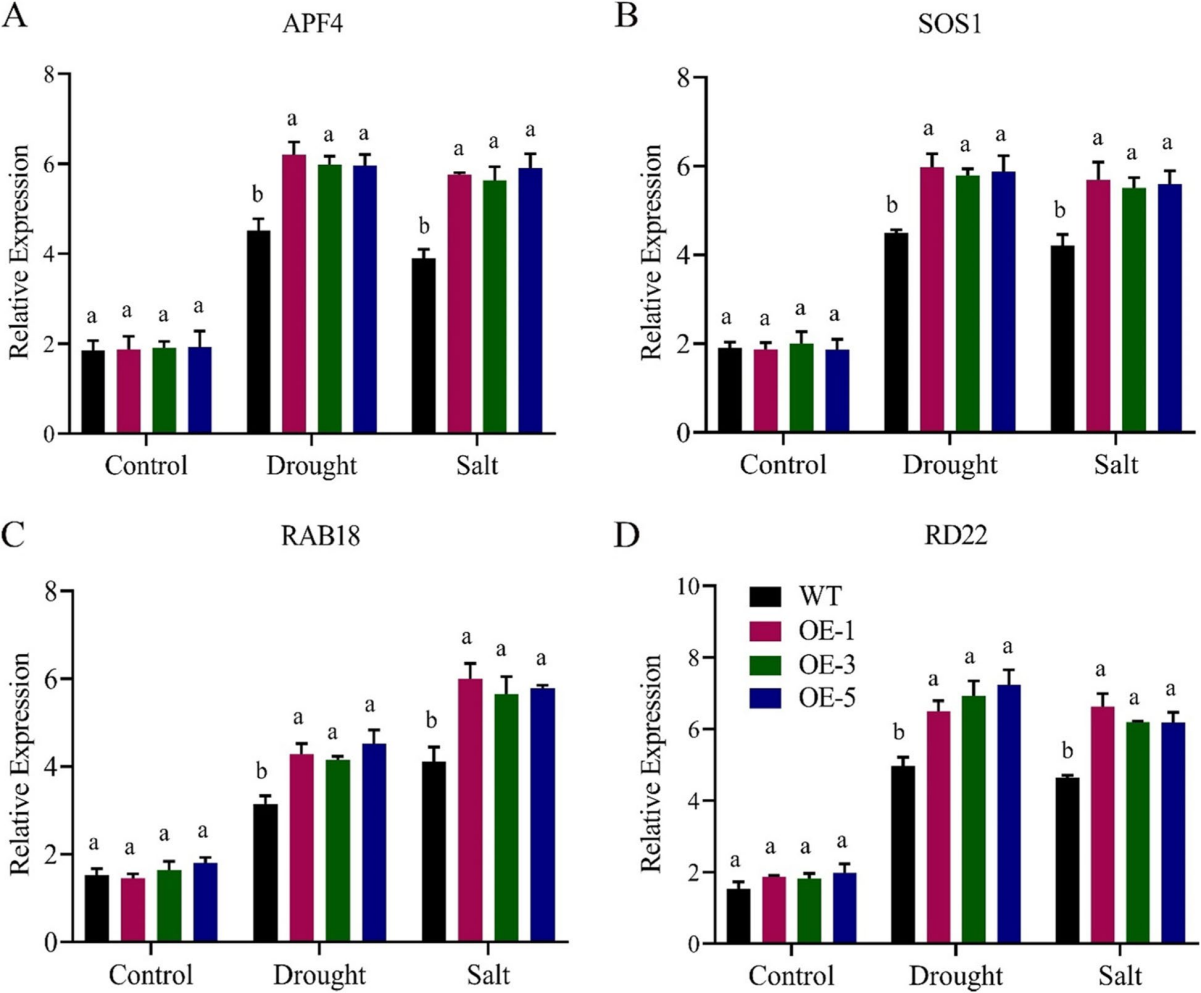
Relative expression analysis of abiotic stress responsive genes **A** *APF4*, **B** *SOS1*, **C** *RAB18*, **D** *RD22*, every experiment had three biological replications., means were calculated using LSD at the *P* < 0.05 and *P* < 0.01 confidence interval

The four responsive genes *APF4*, *SOS1*, *RAB18*, and *RD22* were significantly upregulated during drought and salinity stress situations in the wildtype in the reverse transcription quantitative (RT-qPCR) analysis (Fig. 10). Current results indicate that the overexpression of *GhNAC072* has a valuable effect on the upregulation of stress responsive genes expressions in *A. thaliana*, indicating that regulation of *GhNAC072* might be crucial in the abiotic stress tolerance of crops.

## Discussion

Plants are vulnerable to several types of abiotic stresses, the most notable of which is drought stress. Drought has currently gotten a lot of attention due to its severe impact on crop production [28]. Similarly, salinity leads to a significant loss in crop output every year. The lower osmotic potential of soil solutes in high salinity soils might result in a huge loss of crop production [29, 30].

The *NAC* gene family has 305 genes in *G. hirsutum*, 150 genes in *G. arboreum*, and 153 genes in *G. raimondii*. Both the exons and introns take part in the protein formation. Both follow the DNA packaging mechanism to fit inside a cell. Gene structure arrangements are thus made by comparing exons conferring to their size and reading frame [31]. Exon–intron ratios in *G. hirsutum* and *G. arboreum* were lower, but there was upstream/downstream attachment at the beginning and end of the structures in *G. raimondii*. Many stress responsive genes that play a crucial part in boosting osmotic and salinity stress in cotton have low intron interruption [32]. In the three *Gossypium* species, the distribution of genes was unequal, although six chromosomal pairs were discovered to possess a similar number of genes. Thus, showing the extent of gene loss during the evolution process by observing the contribution of progenitors in developing tetraploid *Gossypium* species throughout evolution [25]. The unequal distribution of genes on the cotton genome's chromosomes could be because of gene repetition or a partial segment of gene duplication that happened during the plant's long evolutionary history. With each duplication, cotton's whole gene sequence was doubled, and the extra genes were recombined or eliminated throughout time [26].

*NAC* genes positively regulate abiotic stress related genes during harsh environmental conditions. Previous studies identified *CaNAC46* in *Capsicum annuum* [33], *ATNAC3* in *Solanum lycopersicum* [34], *PgNACs* in *Pennisetum glaucum* [35] improves drought and salinity tolerance, *AmNAC11* from *Ammopiptanthus mongolicus* boosts drought and cold stress tolerance in *A. thaliana*, *OsNAC2* and *ONAC022*in rice positively controls drought and salinity stress via ABA synthesis mechanisms [11, 12].

In case of physiological traits, the wildtype seedlings had the highest excised leaf water loss and ion leakage after drought and salinity stress treatments relative to the overexpressed lines. Whereas, relative leaf water content and chlorophyll content for the tested treatments were recorded significantly higher in the overexpressed lines. When the crops were subjected to numerous stress situations, the overexpressed lines outperformed the wildtype in terms of stress resistance. The *GhDTX/MATE* transgenic lines scored the highest amount of leaf water content. However, low in leaf water loss and cell membrane stability over the wildtype Arabidopsis seedlings during drought, salinity, and cold stress [36]. Similar to this finding, leaf water content and chlorophyll content were increased, and the leaf water loss and ion leakage exhibited a substantial decrease in overexpressed lines compared with the WT lines were reported in *GhMPK3* genes from cotton [37]. The overexpressed lines had the highest CAT and SOD concentrations but lower levels of MDA and H_2_O_2_ oxidant enzymes in relative to the wildtype in drought and salinity stress environments. This showed that overexpressed lines face low oxidative stress as compared to the wildtype under stress conditions. Under drought and salinity situations, the level of oxidants (MDA and H_2_O_2_) were found to be considerably greater in the wildtype and significantly lower in the transgenic lines. Antioxidant levels in transgenic lines were found to be higher than in wildtype [37]. Alike findings were stated in previous works from *GhTOM* transgenic lines in improving salinity stress tolerance in *A. thaliana* [38].

*GhNAC072* enhances the germination rate and root length capacity of *A. thaliana* during drought and salinity stress treatments. The germination percentage and root length of *GhNAC072* overexpressed lines were higher than that of wildtype under different mannitol and NaCl concentrations. This showed that the *GhNAC072* gene enhances the tolerance ability in *A. thaliana* seedlings during stress conditions. Under long drought and extreme salt stress, the *CaNAC46* transgenic lines improve root length and lateral branches than wildtype [33]. *GmNAC8* transgenic lines demonstrated greater tolerance and survival rates under drought stress, but *GmNAC8* VIGS data suggested the contrary [7]. The profiling of *MeNA*C genes was studied in cassava under drought, ABA, salinity, cold, and H_2_O_2_ conditions, revealing that *NACs* may serve as a site of junction for many signaling pathways [39]. Similarly, functional validation using transformation and silencing of transgenic lines demonstrate that overexpression of *ONAC066* enhanced while knockdown of *ONAC066* diminished oxidative stress tolerance in rice [40]. *GhNAC072* expression profiling with the stress responsive genes*APF4*, *SOS1*, *RAB18*, and *RD22* upregulated during drought and salt stress conditions, signifying that *GhNAC072* may have a crucial role in the abiotic stress tolerance. In line with these results, the accumulation of reactive oxygen species was influenced by *CaNAC46*. Moreover, during drought and salinity stress, *CaNAC46* increased the profiling of responsive genes *RD29B*, *RD20*, *LDB18*, *P5CS*, and *IAA4* [33]. In line with this, in the *mOsNAC2* overexpressing lines, reverse transcription quantitative PCR study revealed a considerable boost in the profiling of the ABA biosynthesis genes *OsNCED1*and *OsNCED3*, as well as the stress responsive genes *OsP5CS1*, *OsLEA3*, and *OsRab16* [11].

## Conclusion

Herewith, we confirmed on the basis of current results that the cotton *GhNAC072* genes plays a significant role in improving drought and salinity stress tolerance. Physiological traits, biochemical parameters, germination rate, and root length evaluation, and stress responsive genes expression analysis showed that *GhNAC072* is a potential involved in boosting tolerance mechanisms under drought and salinity stress. In summary, these results offer data that can be used for applications of *GhNAC072* function in plants and can lead to genetic enhancement of crops in the field of molecular breeding and crop selection.

## Materials and methods

### Planting materials

*Arabidopsis thaliana* Col-0 was used to study overexpression. The seeds were vernalized in dark for two days at 4 °C before being moved to the growth chamber. The plants were grown in a growth chamber with a temperature of 22 °C, a photoperiod of 16 h of light/8 h of darkness, and relative humidity of 80%. Then, the *A. thaliana* seedlings were grown in pots with a 1:1 soil mixture of enriched soil and vermiculite. T0 – T3 seeds were screened, and T3 progeny was used for the final treatment [41].

### NAC family and phylogenetic tree analysis

CottonFGD database (www.cottonfgd.org) was used to identify the NAC genes in cotton species [28]. Using the identified genes from *G. hirsutum* species, the expression analysis of the all genes obtained from the RNA-Seq (NCBI Accession: PRJNA663204) was performed to identify the candidate gene “*GhNAC072*”. The amino acid sequence of the genes of NAC family members in *G. hirsutum*, *G. arboreum*, and *G. raimondii*, were used to develop the phylogenetic tree. The protein sequences of the selected species were aligned via ClustalX and the tree was built by MEGA 7.0 applying the neighbor-joining (NJ) technique under 1000 bootstrap replications [42].

### Chromosome mapping and gene ontology analysis

The GFF3 file from CottonFGD (www.cottonfgd.org) and the gene IDs were used to determine the distribution of NAC TF genes across all chromosomes of A, D, and AD cotton genomes. Then, using TBtools, the gene locations were shown on the chromosomes using data from GTF/GFF3 file. The functional taxonomy of genes were established using an online program AgriGO (www.bioinfo.cau.edu.cn/agriGO), which was based on cellular composition, biological processes, and molecular function [27].

### Gene structure analysis and motif identification

To arrange the gene structure of the *Gossypium* species *G. hirsutum*, *G. arboreum*, and *G. raimondii*, CDS sequences and genomic DNA sequences of the genes were uploaded to gene structure display server (http://gsds.gao-lab.org/) website [43]. Motif identification analysis was performed by uploading the protein sequence of the cotton species to the Multiple Expectation Maximization for Motif Elicitation-MEME website (http://meme-suite.org/tools/meme), thus using the Mast-XML file and gene IDs to visualize the graph via TBtools software [44].

### Co-expression network analysis of genes

We used already available RNA-Seq data of leaf and root tissues under drought stress to check the expression patterns of NAC gene family. The links between genes involved in drought stress tolerance were examined by coexpression network analysis. A coexpression regulation network of the genes linked to drought stress tolerance was created using the Cytoscape software (version 3.7.2) [45]. The threshold for the coexpression network map was set as *p* ≥ 0.99. The topological coefficient of each node with a degree ≥ 20 was used to identify the network as hub genes.

### RNA extraction, cDNA synthesis, and RT-qPCR analysis

Total RNA was extracted from drought and salt treated leaf and root tissues by using a Tiangen (www.tiangen.com) RNA extraction kit. RNA was subsequently reverse transcribed into cDNA using a TaKaRa reverse transcription kit. DNaseI treatment was used to eliminate genomic DNA contamination, following manufacturer procedures. Three biological repeats were maintained throughout the analysis [46]. Real-time quantitative PCR was used to assess the transcript profiling of NAC in leaf and root samples under various stress conditions.The following settings were used to run the PCR: 95 °C for 10 min, which was followed by 45 cycles of 95 °C for 30 s and 60 °C for 10 s. The 2^^CT method of analysis was used and each sample was analyzed in triplicate [47]. *AtActin* was used as the internal reference. The RT-qPCR analysis were carried out three times independently, with three technical repeats for each treatment time point for each experiment [48].

### Plant transformation and screening

Overexpression was performed in *Arabidopsis thaliana* (Colombia-0). The seedlings were grown in a growth chamber with a temperature of 22 °C, a photoperiod of 16 h of light/8 h of darkness, and an 80% relative humidity. T0-T3 seeds were screened, and T3 lines were used for treatment testing. First generation seeds (T1) were collected from T0 generation Arabidopsis seedlings. T1 seeds were sown on media containing Antibiotics and select the segregant lines. Grow the segregant lines and harvest seeds from them for T3 generation. At this stage, we chose lines with 100% purity and do RT-qPCR and PCR for the final selection of the three best lines [37]. From the seven transformed *GhNAC072* lines, we chose OE-1, OE-3, and OE-5. Homozygous lines were used for stress treatment and phenotypic studies.

### Determination of drought and salt tolerance in the transgenic lines

*GhNAC072* overexpressed Arabidopsis lines, OE-1, OE-3, OE-5, and wildtype seeds were sterilized by soaking them in 15% Sodium hypochlorite for five minutes and then rinsing them three times in sterilized deionized water. The seeds were sterilized before being sown on ½ MS media and kept in a dark chamber at 4 °C for two days before being incubated at 22 °C with a 16 h light/8 h dark photoperiod. After 7 days active seedlings were transplanted into pots that have a 1:1 mix of vermiculite and humus. Three weeks later, the plants were exposed to abiotic stresses. Overexpressed lines OE-1, OE-3, OE-5, and WT plants were treated with 20% polyethylene glycol for drought stress treatment, while 200 mM NaCl was used for salt stress treatment. After eight days, physiological and phenotypic traits were measured. The samples were promptly freeze in liquid nitrogen and kept at a temperature of − 80 °C to be used for subsequent gene expression analysis. All sampling were replicated three times biologically and three times technically [49].

### Germination rate and root elongation determination

Under drought and salt stress conditions, the germination rates of overexpressed lines and wild-types were evaluated. Overexpressed lines OE-1, OE-3, OE-5, and wild-type seedlings were sterilized and spread on plates at ½ MS media with mannitol supplementations of 0 mM, 100 mM, 200 mM, and 300 mM for drought simulation. Similarly, to apply salt stress, ½ MS was supplemented with 0, 100, 150, and 200 mM NaCl levels [50]. The germination percentage was measured after 10 days. To simulate drought and salt stress, transgenic and wildtype seedlings were seeded on ½ MS media for 6 days before being transferred to ½ MS supplemented with different amounts of mannitol and NaCl levels. The seedlings were grown for 6 days before having their roots measured on the 7^th^ day after treatment. All measurements were replicated three times biologically and three times technically [51].

### Stress responsive genes expression analysis

The expression levels of four abiotic stress-responsive genes were explored for *GhNAC072* by PCR using specific gene primers to further explore the function of *GhNAC072* under conditions of exposure to drought and salinity stress in cotton. The four stress responsive genes include *ABF4*, *SOS1*, *RAB18*, and *RD22* [52]. Specific primers for these genes were designed using NCBI (https://www.ncbi.nlm.nih.gov/) database (Table S2).

### Statistical analysis

The data were analyzed by one way analysis of variance (ANOVA), and significant differences among the individual means were determined using Least Significant Difference (*P* < 0.05 and *P* < 0.01) at the 5% and 1% confidence levels, respectively. In addition, the graphs were drawn using a PrismPad graph, and the gene family analysis was analyzed and organized using various web tools.

## Supplementary Information

**Additional file 1:** **Supplementary Figure S1.** Chromosomal locations of NAC genes in three cotton species. The chromosomal position of cotton Species were mapped inline to their genome. A, *G. hirsutum* with At subgenome B, *G. hirsutum* with Dt subgenome C, *G. arboreum* D, *G. raimondii* E, Scaffold collections.

**Additional file 2:** **Supplementary Figure S2**. Gene ontology (GO) annotation classification. A, Biological function for *G. hirsutum* B, Molecular function of *G. hirsutum* C, Biological function for *G. arboreum* D, Molecular function of *G. arboreum* E, Biological function for *G. raimondii* F, Molecular function of *G. raimondii*, there is no significant classification for cellular function, the logo stands for the level of significance.

**Additional file 3:** **Supplementary Figure S3.** Phylogenetic relationships of gene structure analysis and Motif Identification in Gossypium species of NAC genes. A, *G. hirsutum*, B, *G. arboreum*, C, *G. raimondii.*

**Additional file 4:** **Supplementary Figure S4.** PCR amplification and gel band formation of the 1038bp coding sequence gene *Gh_D01G0514* (*GhNAC072*) using 5000bp marker.

**Additional file 5:** **Supplementary Table S1.** Number of NAC genes and their assembly in *G. hirsutum*, *G. arboreum*, and *G. raimondii.*

**Additional file 6:** **Supplementary Table S2.** List of primers for cloning, stress responsive genes and RT-qPCR analysis.

**Additional file 7:** **Supplementary Table S3. **Data for germination rate, and root length of transgenic and wildtype during drought and salt stress treatment.

**Additional file 8:** **Supplementary Table S4. **Physiological traits (ELWL, RLWC, CMS and Chlorophyll content) of transgenic and wildtype during drought and salt stress treatment.

**Additional file 9:** **Supplementary Table S5.** Biochemical parameters (SOD, CAT, MDA and H_2_O_2_) of transgenic and wildtype during drought and salt stress treatment.

**Additional file 10:** **Supplementary Table S6.** Data for relative expression of transgenic and wildtype during drought and salt stress treatment.

## Data Availability

The RNA-Seq data of the three *G. hirsutum* lines, Mariegalante-85, Upland cotton and Latifolium-40 under drought stress were found in NCBI (Accession: PRJNA663204). The *Arabidopsis thaliana* Col-0 planting materials used in the experiment came were obtained our laboratory. In addition, additional tables and figures were included as article supplementary files.

## Abbreviations

TF: Transcription factor
RNA: Ribonucleic Acid
CottonFGD: Cotton Functional Genomic Database
WT: Wildtype
LSD: Least Significant Difference
SOD: Superoxide dismutase
POD: Peroxidase
CAT: Catalase
MDA: Malondialdehyde
H_2_O_2_: Hydrogen per oxide
mM: Milli mole
