# Supplementary figures and images for: Overexpression of cotton GhNAC072 gene enhances drought and salt stress tolerance in transgenic Arabidopsis
Source: BMC Genomics. 2022 Sep 12;23:648. doi: 10.1186/s12864-022-08876-z (PMC9469605; doi:10.1186/s12864-022-08876-z)

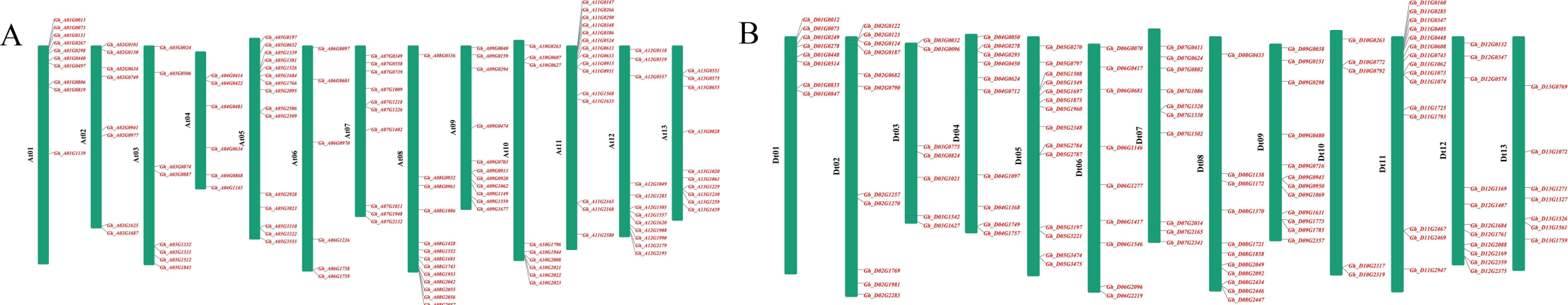

Supplement: Supplementary file 1 — Additional file 1: Supplementary Figure S1. Chromosomal locations of NAC genes in three cotton species. The chromosomal position of cotton Species were mapped inline to their genome. A, G. hirsutum with At subgenome B, G. hirsutum with Dt subgenome C, G. arboreum D, G. raimondii E, Scaffold collections. [file 12864_2022_8876_MOESM1_ESM.pdf]

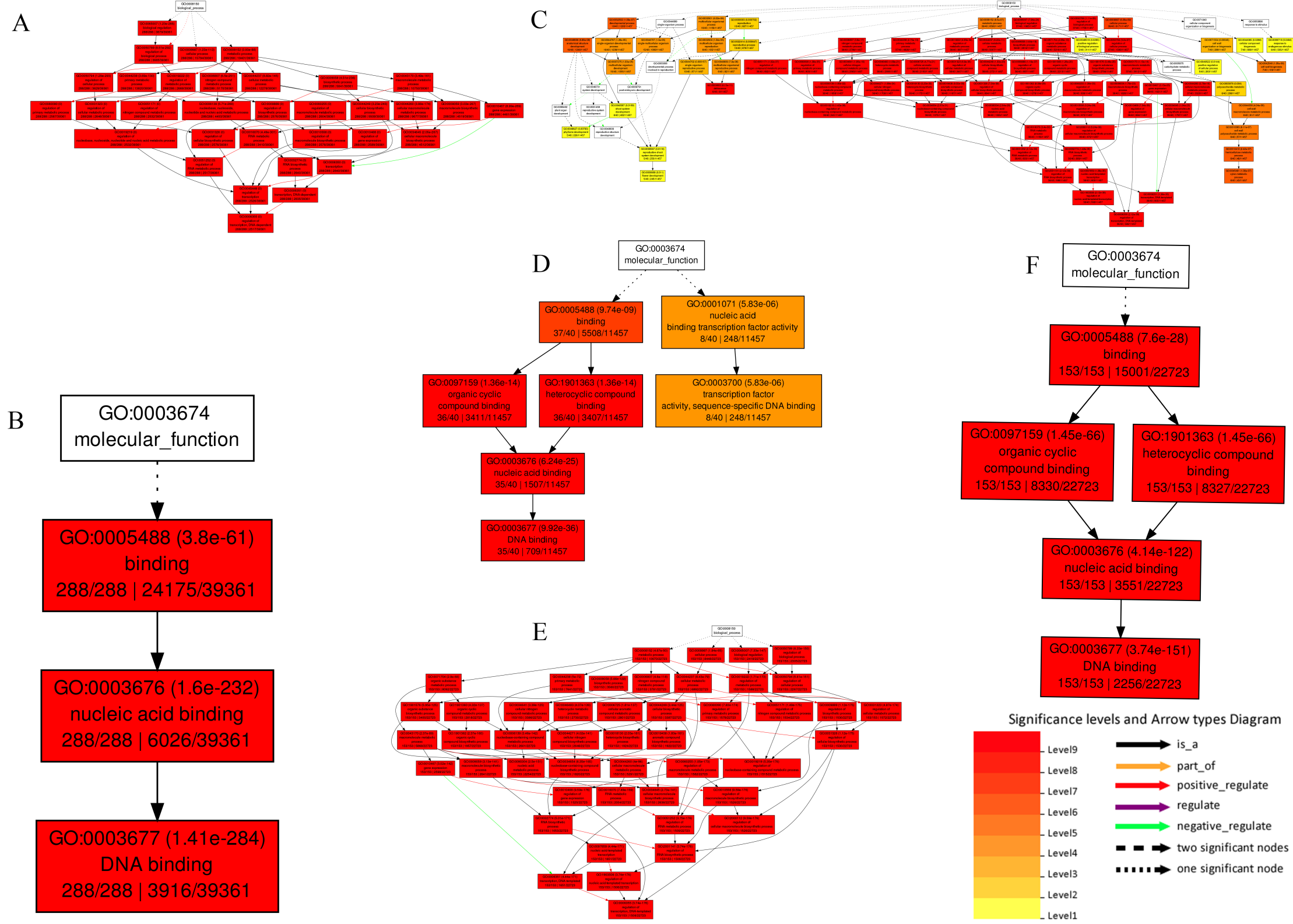

Supplement: Supplementary file 2 — Additional file 2: Supplementary Figure S2. Gene ontology (GO) annotation classification. A, Biological function for G. hirsutum B, Molecular function of G. hirsutum C, Biological function for G. arboreum D, Molecular function of G. arboreum E, Biological function for G. raimondii F, Molecular function of G. raimondii, there is no significant classification for cellular function, the logo stands for the level of significance. [file 12864_2022_8876_MOESM2_ESM.pdf]

A

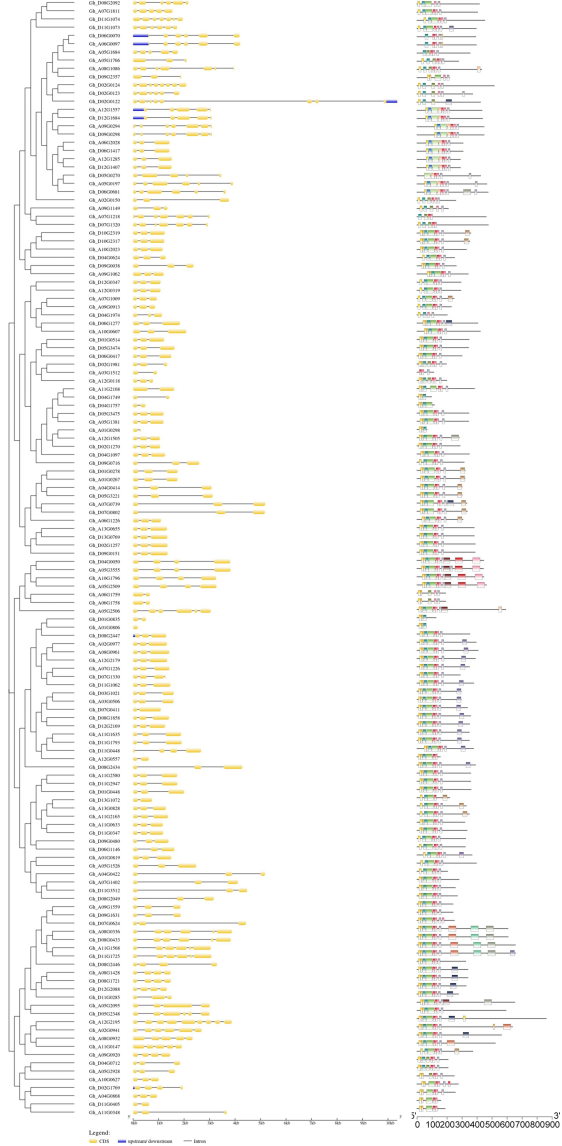

B

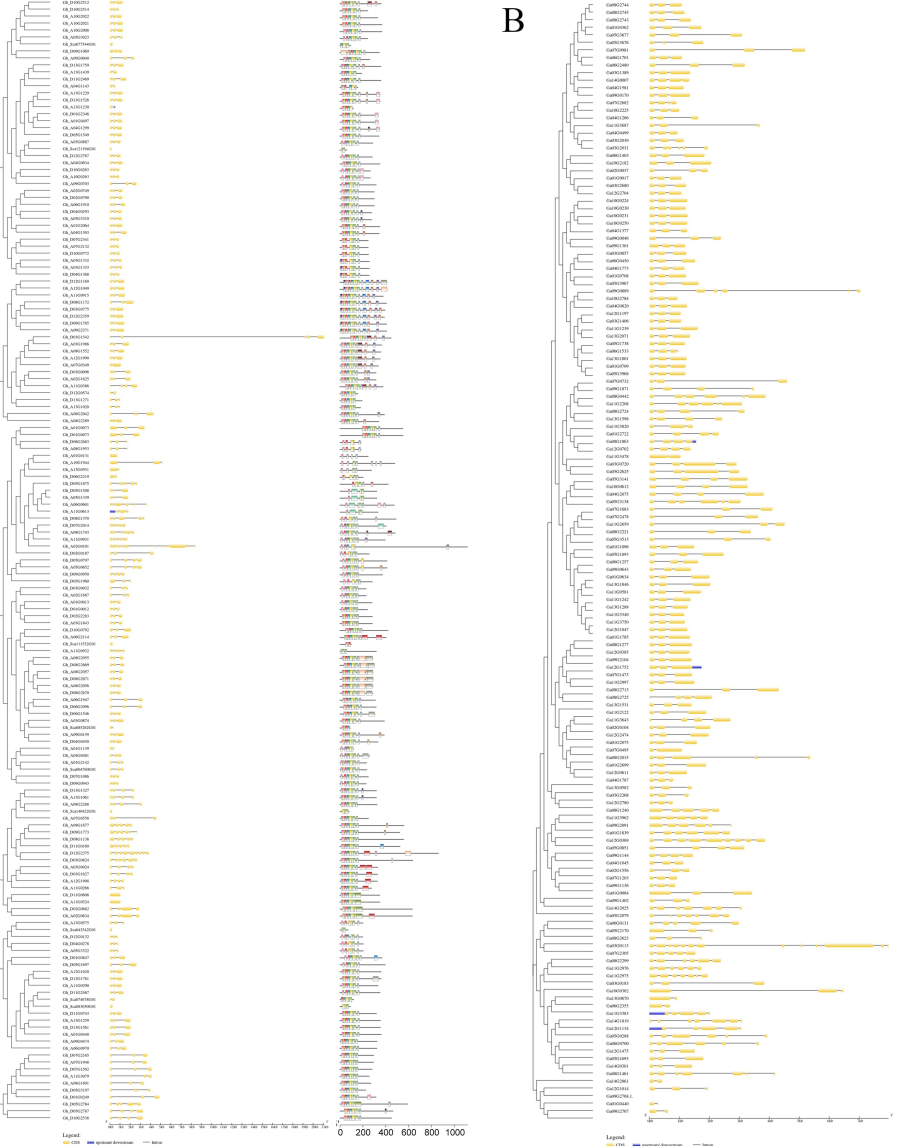

C

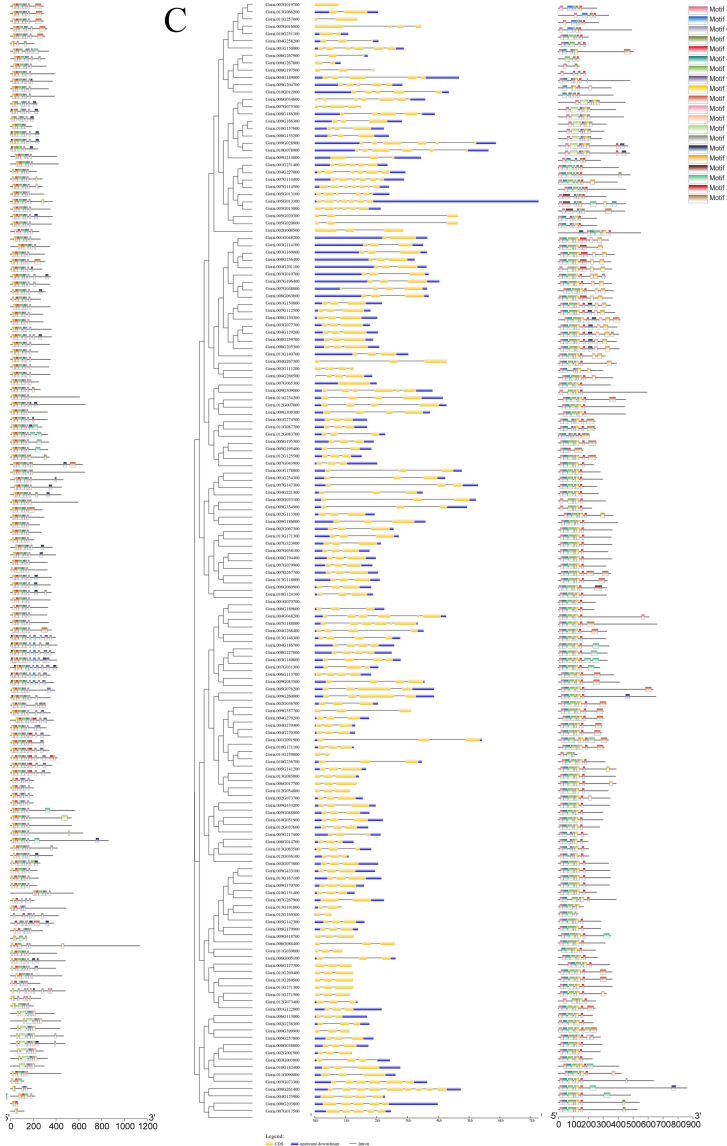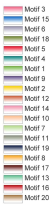

Supplement: Supplementary file 3 — Additional file 3: Supplementary Figure S3. Phylogenetic relationships of gene structure analysis and Motif Identification in Gossypium species of NAC genes. A, G. hirsutum, B, G. arboreum, C, G. raimondii. [file 12864_2022_8876_MOESM3_ESM.pdf]

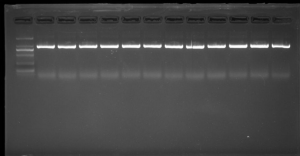

Supplement: Supplementary file 4 — Additional file 4: Supplementary Figure S4. PCR amplification and gel band formation of the 1038bp coding sequence gene Gh_D01G0514 (GhNAC072) using 5000bp marker. [file 12864_2022_8876_MOESM4_ESM.pdf]
